# Editorial: Innovation in Rheumatic Diseases

**DOI:** 10.3389/fmed.2021.801515

**Published:** 2022-01-05

**Authors:** Maria João Jacinto, Pedro Oliveira, Ana Maria Rodrigues, Helena Canhão

**Affiliations:** ^1^Patient Innovation, Lisbon, Portugal; ^2^Copenhagen Business School, Copenhagen, Denmark; ^3^NOVA School of Business and Economics, Universidade NOVA de Lisboa, Lisbon, Portugal; ^4^CHRC, Comprehensive Health Research Center, NOVA Medical School, Universidade NOVA de Lisboa, Lisbon, Portugal; ^5^EpiDoC Unit, Chronic Diseases Research Centre, NOVA Medical School, Universidade NOVA de Lisboa, Lisbon, Portugal; ^6^Rheumatology Unit, Hospital Lusiadas, Lisbon, Portugal; ^7^Rheumatology Unit, Hospital CUF Tejo, Lisbon, Portugal; ^8^Rheumatology Unit, Centro Hospitalar Lisboa Central, Lisbon, Portugal

**Keywords:** innovation, rheumatic diseases, lupus, rheumatoid arthritis, diagnosis, treatment

Rheumatic diseases are high prevalence diseases which affect people of all ages. Inflammatory rheumatic conditions warrant for an early diagnosis and an effective treatment so patients can better manage their disease and its effects. On another hand, osteoporosis and osteoarthritis affect elderly population the most; with the increasing number of older people in countries worldwide, it leads to a major impact on patients' well-being and on society's high economic costs due to healthcare and social resources consumption. Thus, innovation has become a hot topic that aims to solve sustainability of healthcare systems and patients' real needs. In that sense, innovation in rheumatic diseases presents a wide broad of topics, namely novel disease management and treatment approaches, new drugs, therapies, and medical devices, as well as additions to fundamental research.

All these challenging issues justify the edition of our Research Topic “Innovation in Rheumatic Diseases” which, as we expect, can bring up some of the breakthroughs and point out good practices to tackle rheumatic health needs. This group of papers has in common the fact that they cover practical approaches focused on innovation in rheumatic disorders, which include disease knowledge, diagnosis, disease management, well-being improvement, and treatment.

This Research Topic gathers 11 papers from different research groups that deal with distinct aspects of innovation in rheumatic diseases—advances in diagnosis, new mechanisms of disease, innovative technologies, artificial intelligence applications and new contributes to health innovation arena. Eight of them are original research, one clinical trial, one review and one brief research report.

On one side, the rheumatology diseases understanding reveal to be a relevant topic for 4 different research groups. Ingegnoli et al. reviewed the connection between autonomic nervous system and rheumatoid arthritis (RA), where the authors explored common cardiac and mental health-related RA comorbidities with potential relationships to systemic and joint inflammation. Chen et al. performed a population-based study to investigate the relation between anti-phospholipid syndrome (APS) and the risk of newly diagnosed systemic lupus erythematosus (SLE), which showed a significant correlation mainly within the 1st years of APS diagnosis, which highlights the need for vigilance of SLE-associated symptoms in patients recently diagnosed APS. Ma et al. successfully established biomarkers and diagnostic patterns for RA early diagnosis, using serum protein profiles analysis by MALDI-TOF-MS combined with magnetic beads, proteomic analysis and biomarkers identification and quantification, involving patients with different disease activity score (in remission, with low disease activity, with moderate disease activity, and with high disease activity). Finally, Gau et al. investigated the impact of anti-neutrophil cytoplasmic antibodies (ANCAs) on clinical manifestation, organ involvement and outcomes in pediatric-onset systemic lupus erythematosus patients; the authors found an indication that patients with ANCAs tend to have hematuria and an absence of typical renal immunofluorescence histology, but no difference in clinical presentations and treatment outcomes, although further studies are needed to verify those findings.

On another hand, rheumatic diseases diagnosis is also a relevant topic.  Monti et al. howed that color duplex sonography of temporal arteries and large vessels, combined with a fast-track approach, contributed to a substantial reduction of permanent visual loss in giant cell arteritis, one of the most feared complications, by shortening the time to diagnosis and treatment initiation. In a different approach, Cipolletta et al. presented an automatic deep-learning algorithm for informative ultrasound image analysis for assessing metacarpal head cartilage integrity, which may contribute to the enhancement of ultrasound reliability for this task and support beginner sonographers during ultrasound training in the future.

New treatment approaches for different rheumatologic diseases were also highlighted by different researchers. Li et al. presented a cohort study that suggests that adding Chinese herbal medicines to conventional therapy may reduce subsequent risk of hearing loss in RA patients, although prospective randomized trials are still recommended to further support a causal relationship. In a different work, Wu et al. evaluated the positive efficacy and safety of a Chinese medicine formula combined with methotrexate in active RA patients' treatment, in comparison with the combination of methotrexate and leflunomide. Luo et al. compared the efficacy and safety of glucocorticoids combined with cyclophosphamide or mycophenolate mofetil in immunoglobulin G4-related diseases patients.

Regarding strategies to tackle rheumatic diseases-based needs,  Quaresma et al. presented a user-friendly, easy to apply and effective quantitative support tool “OrthoRehab” that can assist physicians/therapists when choosing the most suitable ankle-foot orthosis for each patient with foot dysfunction—one of the most likely consequences of RA. In a different approach comparing to the previous described work,  Jacinto et al. showed that patients and their caregivers also present a significant and high-value innovative profile, since these unusual innovators develop novel solutions to solve their own needs whenever the market isn't providing any answer to it.

We believe the papers published in this Research Topic show a comprehensive vision of relevant aspects of innovation in rheumatic diseases and can help to increase knowledge and awareness around these themes. Enjoy the reading!

## Author Contributions

All authors listed have made a substantial, direct, and intellectual contribution to the work and approved it for publication.

## Funding

The authors are grateful for the funding provided by the Fundação para a Ciência e Tecnologia (FCT) through the project PTDC/EGE-OGE/7995/2020 Healthcare Innovation and Entrepreneurship for Impact: Creating a Resilient, Safe, and Crowd-based Innovation System.

## Conflict of Interest

The authors declare that the research was conducted in the absence of any commercial or financial relationships that could be construed as a potential conflict of interest.

## Publisher's Note

All claims expressed in this article are solely those of the authors and do not necessarily represent those of their affiliated organizations, or those of the publisher, the editors and the reviewers. Any product that may be evaluated in this article, or claim that may be made by its manufacturer, is not guaranteed or endorsed by the publisher.

